# Introduction of the Ribo-BiFC method to plants using a split mVenus approach

**DOI:** 10.1186/s13007-025-01494-2

**Published:** 2026-01-13

**Authors:** Karel Raabe, Alena Náprstková, Janto Pieters, Elnura Torutaeva, Veronika Jirásková, Zahra Kahrizi, Palash Chandra Mondol, Christos Michailidis, David Honys

**Affiliations:** 1 Laboratory of Pollen Biology, Institute of Experimental Botany of the Czech Academy of Sciences, Rozvojová 263, 165 02 Prague 6, Czech Republic; 2 Department of Experimental Plant Biology, Faculty of Science, Charles University, Viničná 5, 128 44 Prague 2, Czech Republic; 3 Department of Genetics and Microbiology, Faculty of Science, Charles University, Viničná 5, 128 44 Prague 2, Czech Republic

**Keywords:** Translation, Ribosome, Translation rate, Bimolecular fluorescence complementation

## Abstract

**Background:**

Translation is a fundamental process for every living organism. In plants, the rate of translation is tightly modulated during development and in responses to environmental cues. However, it is challenging to measure the actual translation state of the tissues *in vivo*.

**Results:**

Here, we report the introduction of an *in vivo* translation marker based on bimolecular fluorescence complementation, the Ribo-BiFC. We combined a method originally developed for the fruitflies with an improved low background split-mVenus BiFC system previously described in plants. We labelled small subunit ribosomal proteins (RPS) and large subunit ribosomal proteins (RPL) of *Arabidopsis thaliana* with fragments of the mVenus fluorescent protein (FP). We tested the Ribo-BiFC method using transiently expressed recombinant ribosomal proteins in epidermal cells of *Nicotiana benthamiana*. The BiFC-tagged ribosomal proteins complemented the mVenus molecule and were detected by fluorescence microscopy, potentially visualizing the close proximity of translating assembled 80S ribosomal subunits. Although the resulting signal is less intense than that of known interactors, its detection points to the functionality of the system.

**Conclusions:**

This Ribo-BiFC approach has further potential for use in stable transgenic lines in enabling the visualisation of translational rate in plant tissues and changing translation dynamics during plant development, under abiotic stress or in different genetic backgrounds.

**Supplementary Information:**

The online version contains supplementary material available at 10.1186/s13007-025-01494-2.

## Background

Translation is one of the fundamental cellular processes, during which proteins are synthesised according to the coding sequence of messenger RNA (mRNA) molecules. In plants, the mechanism of protein synthesis is highly conserved and similar to other eukaryotes [1]. Nevertheless, specific regulatory mechanisms have emerged in plants, particularly during plant development, in response to abiotic or biotic stresses and response to other environmental stimuli such as light or presence/absence of nutrients [1–3] The most recognizable component of the translation machinery is the two-subunit ribosome. Ribosomes are large ribonucleoprotein complexes composed of the small ribosomal subunit (40S) and the large ribosomal subunit (60S). When compared to other eukaryotes, plant ribosomes exhibit their specific differences. For example, the 60S subunit is about 20% smaller than that of mammals [4] and comprises 5S, 5.8S, and 25–26S ribosomal RNAs (rRNA), along with approximately 48 ribosomal proteins (RPs) [5]. Additionally, plants possess a specific P-protein named P3 [6].

Translation regulation triggered by rapid changes in the environment or developmental progress requires the translation rate to change dynamically throughout the plant lifespan. Monitoring translational rate has historically been assessed using the polysome profiling method, where ribosome subunits, monosomes and polysomes in a tissue extract are separated by molecular weight in a sucrose gradient and detected by the absorbance profile of the gradient [7]. The polysome/monosome ratio (PM ratio) is generally used to calculate the translation rate and its changes. Although this method is used to analyse the translational state of the tissue, it requires tissue homogenization and extraction. Additionally, several methods for studying the translation utilize protein synthesis reporters (e.g. Luciferase or photoswitchable FPs) or fluorescently tagged components of the translational machinery [7]. Nevertheless, tracking the translation of targeted transcripts under control of their native or modified promoters and/or UTRs still lacks the overview of the overall translational state [7]. Up to date, no *in vivo* method has been established in plants that enables an assessment of the global translation rate of the cell, tissue or organ and its changes using fluorescence microscopy [7].

One such method for *in vivo* fluorescent visualisation of translating ribosomes was described in the *Drosophila melanogaster* system and was used to track assembled, potentially translating ribosomes in Drosophila neurons [8, 9]. This approach is based on ribosomal proteins labelled with a split FP, YFP or mVenus, used for Bimolecular fluorescence complementation (BiFC). BiFC is a method commonly used for verification of protein-protein interactions. In this approach, however, it is used to label the interaction of 40S and 60S subunits in a way that only assembled 80S ribosomes put the BiFC fragments to sufficient proximity for complementation and fluorescence signal emission upon excitation [10]. Due to the overall conservation of translational machinery in eukaryotes, this method could be possibly implemented in other organisms, including plants. Using BiFC for creating in vivo markers (e.g. to track mRNA) in plants was already reported, where the split-FP fusion proteins label hairpins conjugated into 3’ UTR of one specific transcript [11].

Here, we present the first introduction of the Ribo-BiFC in plant systems. We combined the Ribo-BiFC method described in the *D. melanogaster* system [9] with the improved low-background split mVenus BiFC system reported in plants [12]. We chose one *Arabidopsis. thaliana* RP paralogue based on conservation and overall expression pattern and screened one-vector mVenus-BiFC expression cassettes in *Nicotiana benthamiana* transient expression system. We detected fluorescence complementation of the tested Ribo-BiFC constructs in the transient expression system, demonstrating the Ribo-BiFC as proof-of-concept towards fluorescent marker for visualizing translation *in vivo*. This method has the potential to monitor translation rate in stable transgenic plants and can be applied to study translation dynamics during plant development or in response to stress at the cellular level.

## Methods

### Plant material and cultivation

*Nicotiana benthamiana* plants were used for the transient expression experiments. Seeds were germinated in soil for 10 days and transferred to pots, where they were grown at 24 °C under white light in a greenhouse. Leaves of juvenile 4–5-week-old plants were used for the *Agrobacterium tumefaciens*-mediated leaf infiltration transformation. *Arabidopsis thaliana* ecotype Columbia-0 inflorescences were used for RNA extraction to amplify the CDS sequences of the ribosomal proteins. Seeds of *A. thaliana* were surface sterilised (70% ethanol for 1 min, 20% bleach for 10 min, 5x sterile water wash) and germinated on ½ Murashige Skoog media (2.2 g/L MS basal salts; 100 mg/L myo-inositol; 500 mg/L MES; 0.5 mg/L Nicotinic Acid; 0.5 mg/L Pyridoxine·HCl, 1.0 mg/L Thiamine·HCl) pH 5.7 (adjusted with 0.1 M KOH solution) with 0.8% Agar (Sigma). The seeds were stratified at temperature below 8 °C for 48 h and germinated at 21 °C on long day (16 h light/8 hr of night) in vitro. Seedlings were then transferred to soil (Jiffy tablet) and cultivated in a growth room (22 °C, long day) until flowering.

### YFP and mVenus BiFC design

Selected CDS were obtained from TAIR (Supplementary Table 1), processed by the GoldenBraid 3.0 domesticator software [13] and the generated oligonucleotides were used to create GB parts according to the software protocol. Oligonucleotides for the Ribo-BiFC tags were designed manually to split YFP (NY and CY) and mVenus (NmV and CmV) sequences (Supplementary Table 2). The split YFP was divided into NY (amino acid 1-155) and CY (amino acid 155–240), splitting the YFP β-barrel between the 7th and 8th β-strand, which is a classical way to split the FP in BiFC methods [14]. The mVenus sequence was divided into a large NmV (amino acid 1-210) and a small CmV (amino acid 210–238) part breaking the β-barrel between the 10th and 11th β-strand. This advanced division has been reported to have high specificity and lower background [12, 15]. The BiFC parts were cloned as C-terminal fusions to the CDS sequences. Additionally, we added sequences of specific tags to the FP fragments to enable histochemical protein detection (ct-HA-NY, ct-MYC-CY, ct-Flag-CmV). All oligonucleotides used for domestication are listed in Supplementary Table 3. All fragments designed *in silico* were amplified by PCR with Phusion polymerase (Life Technologies) with proofreading activity. Template for ribosomal proteins CDS was cDNA from *A. thaliana* inflorescences obtained using ImProm-IITM Reverse Transcription System (Promega) from isolated RNA with the RNeasy plant Mini Kit (Qiagen). The split YFP BiFC fragments were domesticated from the pBiFCt-2in1-CC initial BiFC vector [16], while the template for split mVenus BiFC fragments was the mVenus sequence used in [17], and 3xFLAG from the pICSL50007 plasmid from the Golden Gate plant kit [18]. PCR-obtained amplicons of the RPL/RPS fragments were cloned into complete CDS sequences in the pUPD2 vector backbones according to the GoldenBraid restriction-ligation protocol [13]. Chemically competent *E. coli* TOP10α cells were transformed by heat shock with the restriction-ligation reaction. Plasmid-containing colonies were selected by appropriate antibiotics and blue/white selection. All plasmids were isolated using GeneJET Plasmid Miniprep Kit (Thermo Scientific) and verified by restriction enzyme digest reaction and Sanger sequencing (LightRun - Eurofins).

### Assembly of the BiFC expression vector

All domesticated sequences were assembled into transcription units (TUs) controlled by a strong viral sporophytic promoter, Cassava vein mosaic virus promoter (pCsVMV) which is comparable to p35S [19], and the NOSt terminator (Supplementary Fig. 1A, **1B**). All vectors were cloned in the GoldenBraid cloning system with extended set of assembly vectors [20]. We prepared single full TUs in expression clones and continued to the destination vector by their combination according to the scheme (Supplementary Fig. 1). The destination backbone is the GoldenBraid 3.0 binary vector pDGB3 ω2 and the inserts were assembled in one restriction/ligation reaction, using the pDB1 α11 to α14 plus pDB3 α2 system [20]. The α11 position contained the BiFC expression cassette with FP-Nt fragment (pCsVMV::CDS1-NY/NmV::NOSt), while the α12 position contained the BiFC expression cassette with FP-Ct fragment (pCsVMV::CDS2-CY/CmV::NOSt). Then, α13 and α14 positions were filled with 35 bp stuffer (sf) sequences and the α2 position contained the cassette expressing free mCherry (pCsVMV::mCherry::NOSt) that served for the identification of successfully transformed *N. benthamiana* cells. We also created the NY, CY, NmV and CmV parts as CDS sequences to express them freely in the *N. benthamiana* cells as one of the negative controls.

### *Nicotiana benthamiana* transient assays

*Agrobacterium tumefaciens* competent cells (strain GV3101) were transformed by plasmids, colonies were selected on YEB medium supplemented with gentamicin (50 µg/mL), rifampicin (50 µg/mL), and spectinomycin (50 µg/mL) and cultivated at 28 °C for 48 h. Single colonies were inoculated in liquid YEB medium and grown overnight at 28 °C. Liquid cultures were pelleted by centrifugation (5 min at 1620 g), washed twice, re-suspended, and diluted to an OD^600^ of 0.2 with infiltration medium (10 mM MES pH 5.6, 10 mM MgCl_2_ and 200 µM acetosyringone). A suspension of *A. tumefaciens* cells carrying the plasmid with an expression cassette of p19 suppressor of silencing was added in an 1:1 ratio of OD^600^ [21]. Mixed suspensions were incubated in dark environment with moderate shaking for 3 h at room temperature and subsequently injected into the abaxial side of 4- to 5-week-old *N. benthamiana* leaves using a 1mL or 2mL syringe. Three days after infiltration, tobacco epidermal cells were analysed microscopically.

### Microscopy and image analyses

Signal of YFP and mVenus was detected in pavement cells of *N. benthamiana*. Imaging was performed by the inverted fluorescent confocal microscope (Axio Observer Z1) Zeiss LSM880 (Carl Zeiss, Jena, Germany) with Plan-Apochromat 20×/0.8 DIC M27 objective. Green fluorophores were excited by the Argon ion laser at 488 nm and the emission range of 508–543 nm was collected on a GAsP detector. The mCherry was excited by the DPSS laser at 561 nm and the emission range of 600–620 nm was collected on a GAsP detector. Representative images were selected and processed identically in ZEN blue software by z-stack selection and maximum intensity projection (Carl Zeiss, Jena, Germany). Single focal planes of different transformed cells were used for quantifications of YFP and mVenus comparison. Presented data are from two of three independent experiments showing similar trends. Nucleoli details of RP-mVenus were imaged with alpha Plan-Apochromat 100×/1.46 Oil DIC.

Imaging for Ribo-BiFC quantification was performed with adjusted setup for lower overall signal strength. Acquisition was performed by the inverted fluorescent confocal microscope (Axio Observer Z1) Zeiss LSM880 (Carl Zeiss, Jena, Germany) with C-Apochromat 40×/1.2 W Korr FCS M27 objective. The mVenus was excited using the Argon ion laser at 514 nm and the emission range 516–570 nm was collected. Single focal planes of different transformed cells were used for Ribo-BiFC quantifications. Presented data are from one of two independent experiments showing similar trends. Nucleolar details of Ribo-BiFC were cut from the image collection for quantification.

Quantification was done on single frame images of at least seven different transformed cells, following a standardized protocol applied to all images, performed in Fiji (ImageJ) [22]. For each image with a transformed cell, a single focal plane was acquired for quantification, and a duplicate was generated on one region of interest selected per image. Regions of interest were manually defined to encompass mCherry-positive cytoplasmic areas while carefully excluding nuclei. The duplicate was then smoothed with a Gaussian filter (σ = 8 pixels) to reduce noise. Huang’s automatic thresholding algorithm was applied to produce a binary mask, which was refined by three successive erosions to remove minor artefacts. Finally, the intensity and shape properties were measured on the original image based on the dedicated mask. Each data point represents mean intensity of the cytoplasmic capture. Considerable data variation caused deviation from normality and application of a non-parametric two-tailed, unpaired Mann-Whitney test for statistical evaluation.

### Protein structure and gene expression visualization

Structural visualization was performed with Chimera 1.15 software [23]. For ribosomal protein visualization and positioning within the 80S structure, we used the structure of plant 80S translating ribosome from *Triticum aestivum* (PDB code: 4V7E) [24]. The YFP structure was visualized from template of the crystal structure of eYFP (PDB code: 6VIO) and the visualization of mVenus FP was created by the template structure of mVenus (PDB code: 6SM0). The expression analysis was performed using Genevestigator^®^ [25] which uses microarray expression data and showed expression levels for each accession according to the plant anatomy or developmental stage. We based our expression analysis on the Affymetrix GeneChip data visualization using the development functions of the Genevestigator^®^ software. For comparison of Arabidopsis RPs, the protein sequences were downloaded from the TAIR database and aligned with the MUSCLE algorithm.

## Results and Discussion

### Split mVenus BiFC shows lower background noise than split YFP

**Fig. 1:**
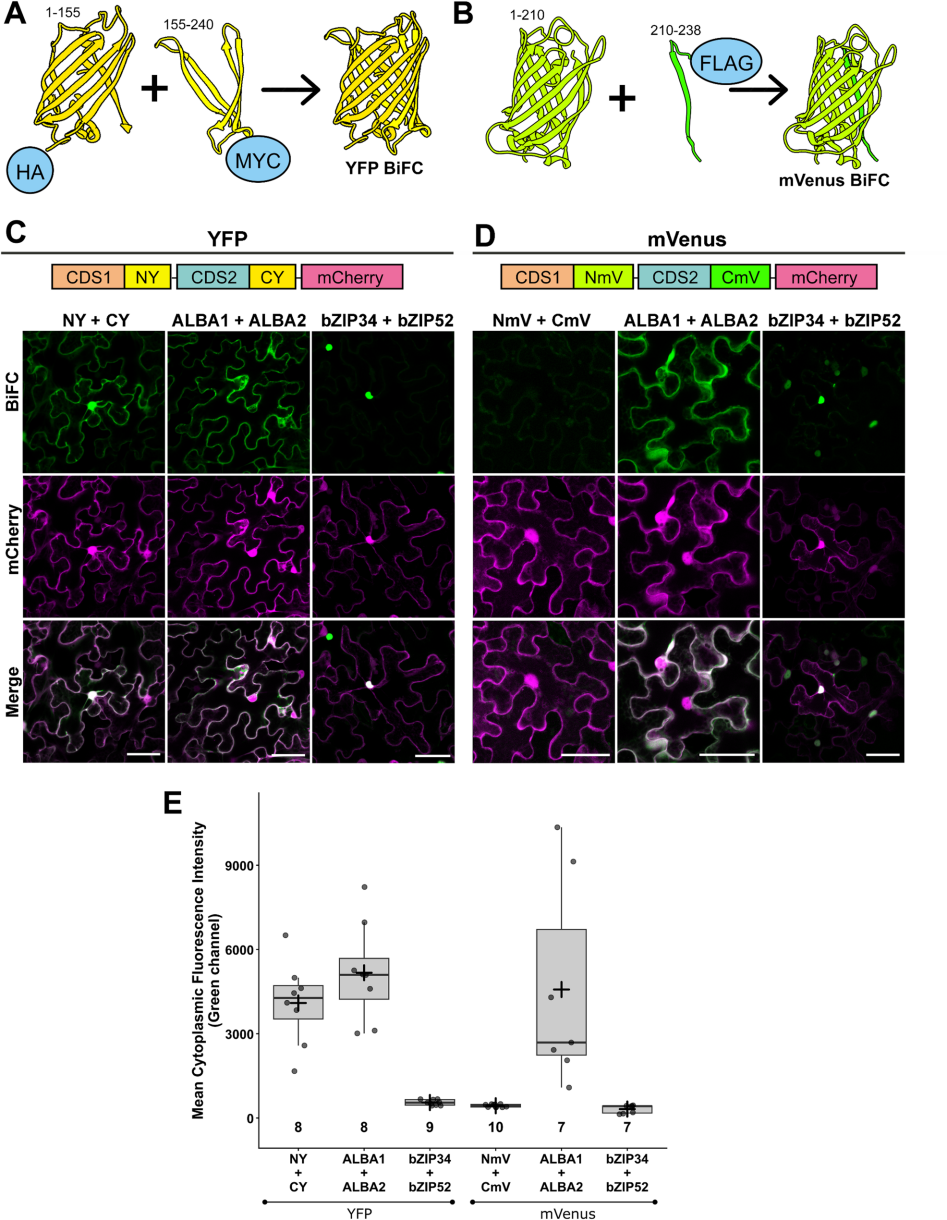
Design of the Ribo-BiFC system and comparison of the split YFP and the split mVenus.Visualization of the BiFC design, depicting **A** the split YFP molecule and **B** mVenus. Numbers indicate amino-acid range of each part of the YFP (NY 1-155; CY 155–240) or mVenus (NmV 1-210; CmV 210–238). Histochemical tags are displayed fused to the N-terminal ends of the respective BiFC fragments (HA-YN + MYC-YC and NmV + FLAG-CmV). Transient co-expression of schematically shown insertion cassettes **C** YFP and **D** mVenus BiFC controls is displayed. The detected signal of free FP BiFC fragments is shown in the first columns of each section (NY + CY and NmV + CmV) in transiently transformed tobacco pavement cells. Signal of known interacting partners ALBA1 + ALBA2 and bZIP34 + bZIP52 was detected and served for the YFP and mVenus BiFC comparison. Data for each sample were obtained by detection of YFP/mVenus signals in green (top row) and a free mCherry signal (middle row) in magenta. The merged channel display overlays of the BiFC samples with the mCherry signal. Scale bar equals to 50 μm. **E** BiFC controls quantification of mean cytoplasmic fluorescence intensity. Boxplots show median (center line), interquartile range (box), and whiskers. Individual data points are overlaid, mean values indicated by +. Numbers indicate sample size (n)

Bimolecular fluorescence complementation (BiFC) is a method developed to visualise interaction between two proteins when they are in proximity. A fluorescent protein is divided into two parts, with each part attached to a different target protein. When the proteins end up in proximity of around 7 nm [26], it results in the assembly and structure restoration of both FP parts and fluorescence emission upon excitation [14, 27]. The Ribo-BiFC developed for *Drosophila* showed that close proteins in the assembled ribosome can also be visualised using the BiFC system. Two FPs, YFP and mVenus, were used in our plant-adapted method to balance the strength of the split FP interaction and reduction of background noise (Fig. 1A, B).

The transient expression of the BiFC controls in *N. benthamiana* showed a clear difference in the split YFP and mVenus molecular tags (Fig. 1). While the expression of the commonly used free YFP parts (NY + CY) showed non-specific complementation and signal emission in the cytoplasm and nucleus, the free parts of mVenus210 (NmV + CmV) revealed a lower background signal, almost comparable with the set of negative controls of p19-transformed, mock-transformed (infiltration media only) and non-transformed cells (Supplementary Fig. 2). We also tested known interacting partners, ALBA1 + ALBA2 [28], as positive controls with a strong signal in the cytoplasm and two dimerizing members of the bZIP family transcription factors that localize in cytoplasm but form dimers in nucleus, bZIP34 + bZIP52 [29, 30], with similar result in both BiFC approaches (Fig. 1C, D). Moreover, the identical complemented signal pattern corresponds with our previous localization of the ALBA family proteins in cytoplasm [31, 32] and bZIP proteins in the nucleus, further confirmed by the quantification of the cytoplasmic signal (Fig. 1E). Altogether, we concluded that the mVenus BiFC is superior to the YFP tag in our transient system.

### Selection of ribosomal proteins for the Ribo-BiFC

**Fig. 2:**
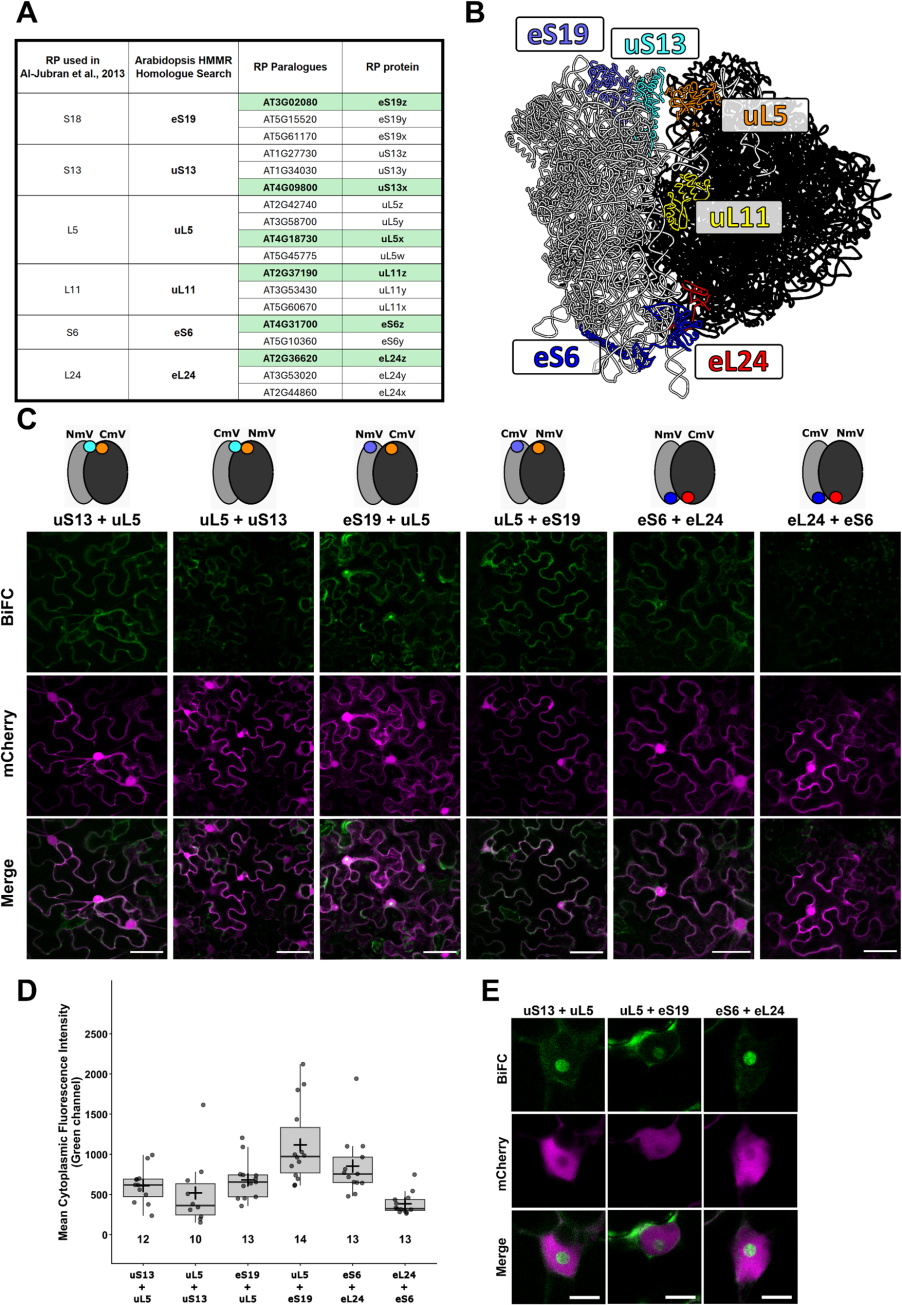
Ribo-BiFC protein selection and proximal combinations in* Nicotiana benthamiana*. **A** Summary of the selection of RPs for Ribo-BiFC testing in plants. In the left part, a RP list chosen for *Drosophila melanogaster* publication is listed and the RP with a similar position within the 80S *Triticum aestivum* model is listed next to it. The right part lists all paralogue genes in *Arabidopsis thaliana* and their corresponding protein names, with the chosen paralogue used in Ribo-BiFC highlighted. **B** The structure of the 80S translating ribosome from *Triticum aestivum* (PDB code: 4V7E) was visualized in Chimera software. Selected ribosomal proteins are highlighted in various colours. Both rRNA and the rest of RPs are coloured in black or grey for 60S and 40S, respectively. **C** Transient *N. benthamiana* assay of promising Ribo-BiFC reciprocal combinations in optimal distance. The upper cartoon section depicts the position and C-terminal tagging with the mVenus BiFC fragment. Representative images of the signal detection are shown in the green channel for mVenus BiFC. Free mCherry control of the pavement cells transient transformation is in the middle (shown in magenta) and the overlay of the channels is displayed in the third row. All transcription units are driven by the pCsVMV promoter and acquired channels were processed in the same way. **D** Ribo-BiFC quantification of mean cytoplasmic fluorescence intensity. Boxplots show median (center line), interquartile range (box), and whiskers. Individual data points are overlaid, mean values indicated by +. Numbers indicate sample size (n). **E** Detailed view on the BiFC signal in the nucleolus of transformed pavement cells. Scale bars equal to 50 μm (**C**) and 10 μm (**E**)

This plant-based Ribo-BiFC for the *in vivo* visualisation of assembled 80S ribosomes is based on the *Drosophila* Ribo-BiFC approach [8, 9]. There, the tagging of multiple small and large ribosomal protein pairs (combinations of two *Drosophila* ribosomal proteins; S18, S13, L5, L11, S6 and L24) was used **(**Fig. 2A, B**)**. To identify the *A. thaliana* orthologues of the RPs in *Drosophila*, we used the assembled 80S ribosome structure from *Triticum aestivum* [24] to identify RPs with a similar position within the ribosome. We then used the RP amino acid sequences from the *T. aestivum* structure for HMMR homology search [33] in *A. thaliana* protein database with standard settings. After identifying each corresponding RP in *A. thaliana*, we searched for its paralogue accessions according to the ribosomal protein gene list [1, 34]. All the selected RPs had multiple paralogous genes in Arabidopsis (Fig. 2A). We evaluated the homology of RP amino acid sequences of the paralogous genes (Supplementary Fig. 3) and their expression profiles (Supplementary Fig. 4). We observed high levels of homology between the paralogs, with sequence identity reaching between 90 % and 100 %. The only exception was the eL24x gene AT2G44860 which shared only around 33 % sequence identity with the other two eL24 genes. Therefore, the gene was excluded from the final RP selection. Protein sequence comparison did not indicate on one preferred paralogue within the gene family. Therefore, we based our choice on gene expression. Ribosomal protein genes with overall high expression and co-expression with other RPs in the plant development were used for Ribo-BiFC tagging – eS6z (AT4G31700); uS13x (AT4G09800); eS19z (AT3G02080); uL5x (AT4G18730); uL11z (AT2G37190); eL24z (AT2G36620). According to their 80S position, we identified optimal pairs for Ribo-BiFC as either eS19 or uS13 paired with uL5 and eS6 paired with eL24 (for simplicity, we omit the paralogue coding in the RP name in text below). From this perspective, we hypothesized that uL11 could also form interaction with uS13/eS19 but with lower efficiency than the optimal pairs. This design also allowed tagging RPs that are on opposite sides within the 80S ribosome, potentially representing a non-optimal combination that should exhibit reduced detection of the BiFC signal in the experiments, which was the case in the *Drosophila* system [8]. According to this perspective, we hypothesized that when 80S ribosomes are assembled during translation, it would ensure that the selected RPs are brought within proximity of each other. These RP pairs, tagged with the split mVenus would serve as a nucleation core for the necessary stabilisation and orientation of the FP parts into the correct conformation and light emission. Confocal microscopy of selected plant tissues could then be performed to visualise the fluorescence intensity that reflects the number of assembled ribosomes. Although we initially tested some of the Ribo-BiFC combinations with both YFP and mVenus system with similar results, we only show here the mVenus Ribo-BiFC.

### Ribo-BiFC in* Nicotiana benthamiana* pavement cells

The testing of the selected Ribo-BiFC pairs showed promising results in the fluorescence signal observation. Overall, the signal strength was much lower than our positive mVenus BiFC control, the ALBA proteins. Additionally, variability in signal strength was observed between individual transformed cells, which is, however, common for transient systems. Given that only assembled ribosomes with both protein fusions integrated can complement the FP emission, the reduced signal strength is expected to be less efficient than cytoplasmic protein-protein interactions.

The selected Ribo-BiFC pairs signal intensity mostly corresponded to the position of the RP pair on the ribosome in most of the cases (Fig. 2C, D, Supplementary Fig. 5). We detected cytoplasmic mVenus signal in Ribo-BiFC pairs uS13 + uL5, eS19 + uL5 and eS6 + eL24 that are in proximity of the two assembled ribosome subunits (within the described pair, first RP is tagged with NmV and the latter with CmV). We also detected fluorescent signal in the reciprocally tagged pair uL5 + eS19. Interestingly, the signal of reciprocally tagged uL5 + uS13 and L24 + eS6 was lower. In comparison, we observed low signal in the sub-optimal Ribo-BiFC pairs, uS13 + uL11 and eS19 + uL11. Additionally, we observed some signal in the RP pair eS6 + uL5 placed on the opposite site of the 80S ribosome, although it was weaker than the optimal pairs described above. We have not observed such signal in the reciprocally tagged uL5 + eS6. These observed differences support the hypothesis that Ribo-BiFC fluorescence complementation is dependent on the RP pairs proximity, however, the suboptimal pairs can produce some complementation, which was also reported in the original *Drosophila* study [8]. Similarly, the differences in signal strengths of reciprocally tagged RP pairs were observed in the original study as well [8]. This pattern may reflect some degree of ribosome proximity that does not correspond strictly to single 80S assembly such as possible contacts of multiple assembled ribosomes on one mRNA molecule, or it may arise from another biological artefact.

From our pre-selected RP pair candidates, the most promising is the combination of eS19 and uL5, which showed higher signal detection in both tested combinations. The positioning of these RPs is also close to the chosen pair in the *Drosophila* RP pair [8] preferred for later studies [9]. As we aimed to test the functionality of the system in plants, we did not test the full scope of all combinations or test the N-terminal RP protein fusions for each of the RP pairs, as the analysis in *Drosophila* also reported mild differences between differently tagged pairs [8]. As the next step is to introduce Ribo-BiFC to stable lines, further combinations of tagging should be included for the promising RP pairs in the future method expansion.

The mVenus signal in Ribo-BiFC samples was also detected in nucleoli (Fig. 2E). The signal in nucleoli may reflect the RP accumulation in this compartment where ribosomes are assembled, as the high number of ribosomal protein fusions produced under the viral promoter is excessive to the number of ribosomes being assembled in the nucleolus. Additionally, protein fusions may be less preferred for incorporation in comparison to the native proteins. Another possible explanation is it may be due to some pre-ribosome assembly occurring in the nucleus. We tested the localization of selected RPs fused to full CDS mVenus on their C-terminal (Supplementary Fig. 6). These constructs showed a similar pattern of localization. The mVenus signal was detected in the cytoplasm, nucleus and particularly in the nucleolus. Similar localization was reported in other ribosomal protein studies in *Arabidopsis* [35] or rice [36] and similar observation of nucleus signal was described in the *Drosophila* Ribo-BiFC [8]. The assembly of ribosomes is hierarchical process and choosing late-assembly RPs could improve avoiding the nucleolus signal in the Ribo-BiFC. In our combinations, the eL24 is such late-assembly protein that should be incorporated in ribosomes at the final stages of 60S maturation, at least in yeast [37]. However, we still observed nucleoli signal in the Ribo-BiFC combination with eS6 (Fig. 2E). To summarize, the revealed Ribo-BiFC signal pattern matches the localization of the RPs. Our hypothesis is that the signal presence in nucleolus is an artefact due to the overexpression rather than a visualization of assembled ribosomes. Use of weaker promoters in the stable lines is therefore preferred for the next testing of the Ribo-BiFC.

To exclude the possibility of a non-specific complementation, we chose to use the uL5 and eS19 proteins to be tested in a set of BiFC controls (Supplementary Fig. 7). We tested combinations of the free NmV and CmV in pairs with either uL5 or eS19 tagged with the complementary mVenus fragment. In these set of controls, we detected some signal in BiFC combinations of ribosomal protein and the NmV BiFC part (NmV + uL5-CmV and NmV + eS19-CmV) in the nucleus (Supplementary Fig. 7A) and cytoplasm, which was still significantly lower than the Ribo-BiFC combinations of these proteins (Supplementary Fig. 7B). We additionally tested the bZIP52 transcription factor with uL5 or eS19 in similar way to test possible non-specific interactions in cytoplasm. These combinations showed no increase in cytoplasmic signal (Supplementary Fig. 7). Occasionally, cells showed weak signal in nucleus in the bZIP52 + eS19z combination. This observation further supports our hypothesis that the nuclei Ribo-BiFC signal may be an overexpression artefact and/or also points to some limitations of the improved split mVenus BiFC approach.

## Conclusions and future perspectives

Here, we describe the Ribo-BiFC proof-of-concept method in plants. This approach is based on the previously reported *D. melanogaster* Ribo-BiFC method, which involves tagging small and large ribosomal proteins with BiFC fragments to visualise their proximity when ribosomes assemble during translation. To improve signal specificity, we have utilized a novel BiFC approach using mVenus FP split setup that reduces the non-specific BiFC signal. We designed the split mVenus fragments in the GoldenBraid system which enables the assembly of all Ribo-BiFC parts into single vector. Further, we tested several candidate pairs of *A. thaliana* ribosomal proteins in *N. benthamiana* transient assays. We were able to detect the mVenus signal in Ribo-BiFC pavement cells that corresponded to the positions of the tagged RPs on the 80S ribosomes, although low amount of signal was present in the distant combinations. The Ribo-BiFC also showed a hypothesized artefact signal localized in the nucleolus, which may be due to overexpression and accumulation of the RPs in this compartment.

The observed signal variability in the transient system is also a limiting factor in quantifying the differences under treatments. The overall variability and weak signal of the Ribo-BiFC could likely be a feature of the translation machinery dynamics, complexity of the ribosome assembly and competition between RP protein fusions and native RPs. Additionally, it is challenging to evaluate the number of copies inserted in the individual cells. Therefore, to overcome some of the mentioned limitations, we aim to produce stable Ribo-BiFC lines of *Arabidopsis thaliana* with some of our tested RP pairs. This establishment would be crucial for advanced experiments and treatments that would confirm the Ribo-BiFC method as a functional translation marker in plant systems. Such treatments will include environmental stresses and translation-drug treatments.

As we have tested similar combinations as in the original report, we would like to further test the pair eS19 and uL5 with consistent signal, in stable lines. It is possible that another RP pair in plants is more suitable for the Ribo-BiFC, we suggest that confirming the Ribo-BiFC reacts to treatments with the proposed combination is adjacent to search for more ideal RP pair in plants at this moment. To further overcome some of our observed limitations, we would aim to test more native conditions in the stable lines, which exclude the use of strong viral promoter that causes free RPs accumulation in nuclei. We suggest achieving more native expression of the RPs either by UBQ10 promoter or native promoters of the used RPs. Other improvements lie in testing all combinations of N- and C-terminal fusions and the use of more flexible linkers rather than histochemical tags. Alternatively, employing other *in vivo* interaction system like FRET is possible with cautionary design of the FP distance within the ribosome.

Consequently, the Ribo-BiFC stable line would permit non-invasive assessment of the translational rate of cells or tissues in different genetic backgrounds or under various environmental or biological conditions. Considering the conserved structure of the translational machinery, this approach has a potential to be introduced in other plant species as well and bring new insights into one of the most dynamic and essential molecular processes.

## Supplementary Information

Supplementary Material 1.

## Data Availability

The materials and data used during the current study are available from the corresponding authors on request.
