# Supplementary material for: Introduction of the Ribo-BiFC method to plants using a split mVenus approach
Source: Plant Methods. 2026 Jan 13;22:17. doi: 10.1186/s13007-025-01494-2 (PMC12888511; doi:10.1186/s13007-025-01494-2)

## Supplementary Figures

**
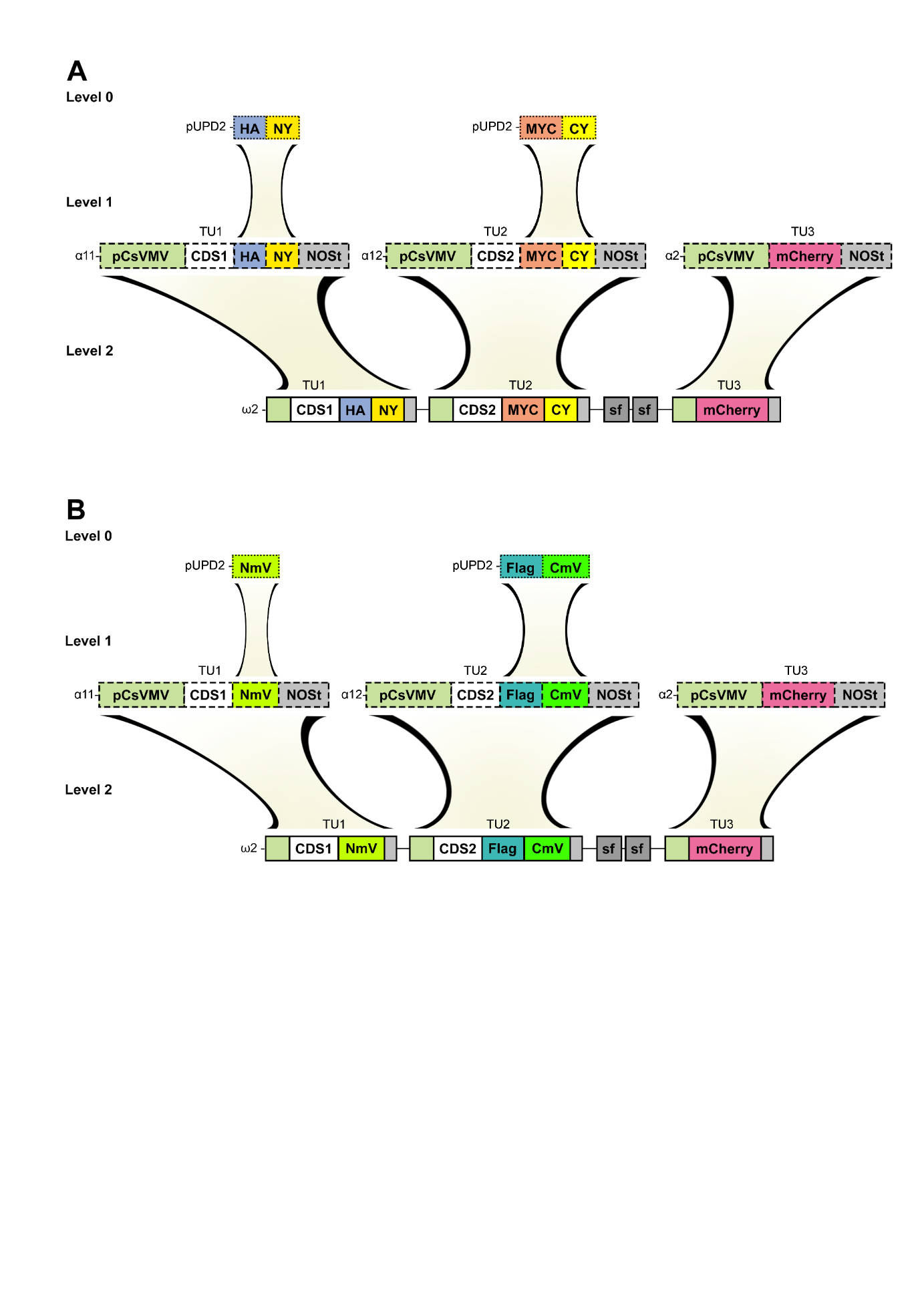
**

**Supplementary Figure 1: Schematic overview of the destination vector assembly**

The individual steps were based on the GoldenBraid 3.0 cloning. **(A)** At the Level 0, fragments of the NY and CY were domesticated from the pBiFCt-2in1-CC vector and N-terminally fused with HA or MYC tags domesticated from MoClo. The selected RPs coding sequences were domesticated according to [13]. Next, the transcription units were assembled at Level 1 and combined with the destination vectors at Level 2. **(B)** Fragments of NmV and CmV were domesticated from the complete mVenus sequence obtained from [17]. While NmV size is sufficient for the GFP antibody recognition, CmV was N-terminally fused with a 3xFLAG tag. Both domesticated split mVenus parts were canonically implemented into Level 1 and Level 2 GoldenBraid cloning.

**
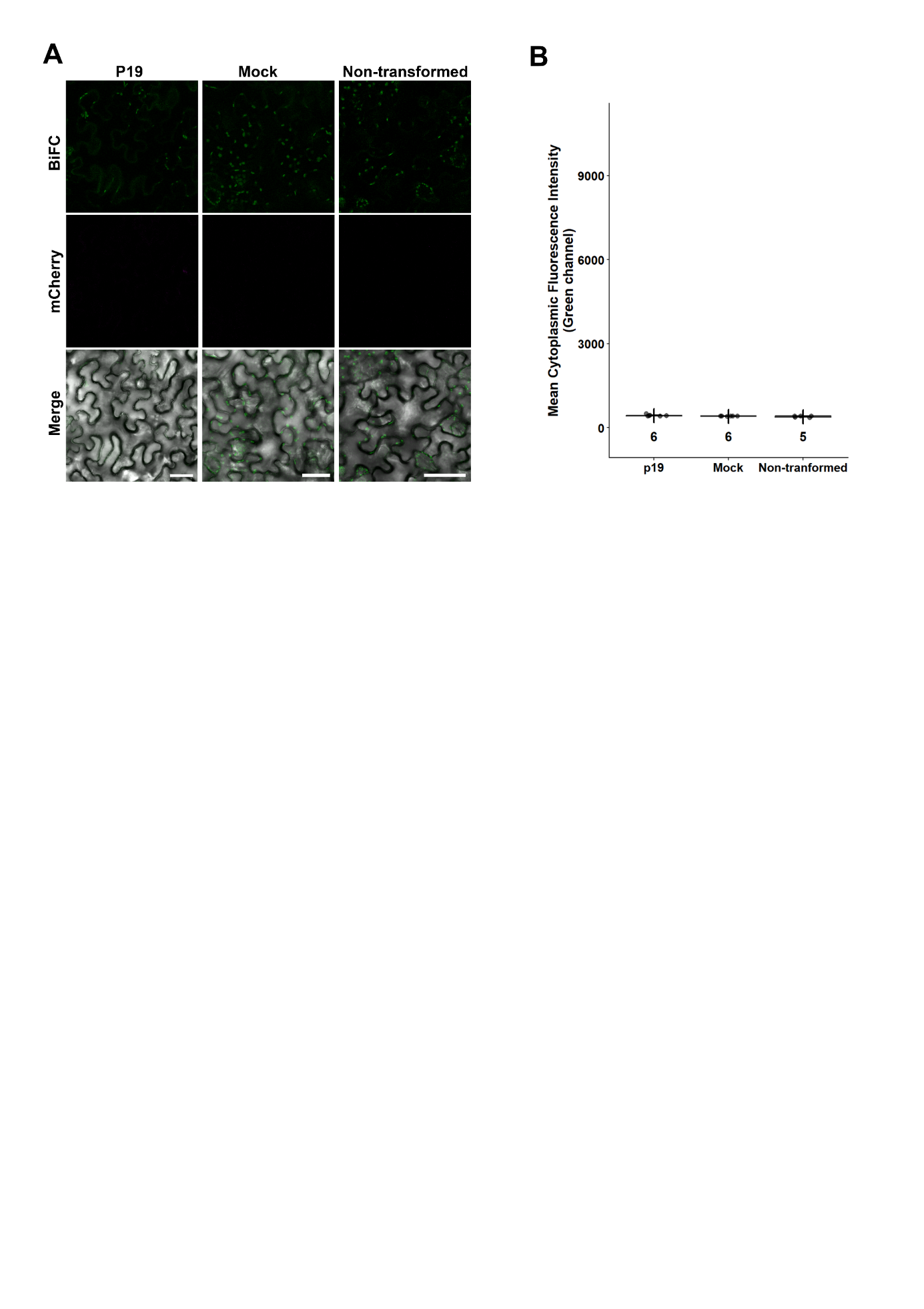
Supplementary Figure 2: Microscopy imaging of background controls in *N.* *benthamiana***

**(A)** Transient *N.* *benthamiana* assay of negative transformation controls. The image was obtained from pavement cells transformed with *Agrobacterium* containing the P19 enhancer plasmid, Infiltration media only and from non-transformed plants. Signal detection is shown in the green channel, red channel and merged image, including the brightfield for mVenus BiFC. Free mCherry control of the pavement cells transient transformation is in the middle (shown in magenta) and the overlay of the channels with the brightfield image is displayed in the third row. Scale bars are equal to 50 µm. **(B)** Mean cytoplasmic fluorescence intensity of the controls. The quantification shows similar background values. Boxplots show median (center line), interquartile range (box), and whiskers. Individual data points are overlaid, mean values indicated by +. Numbers indicate sample size (n).

**
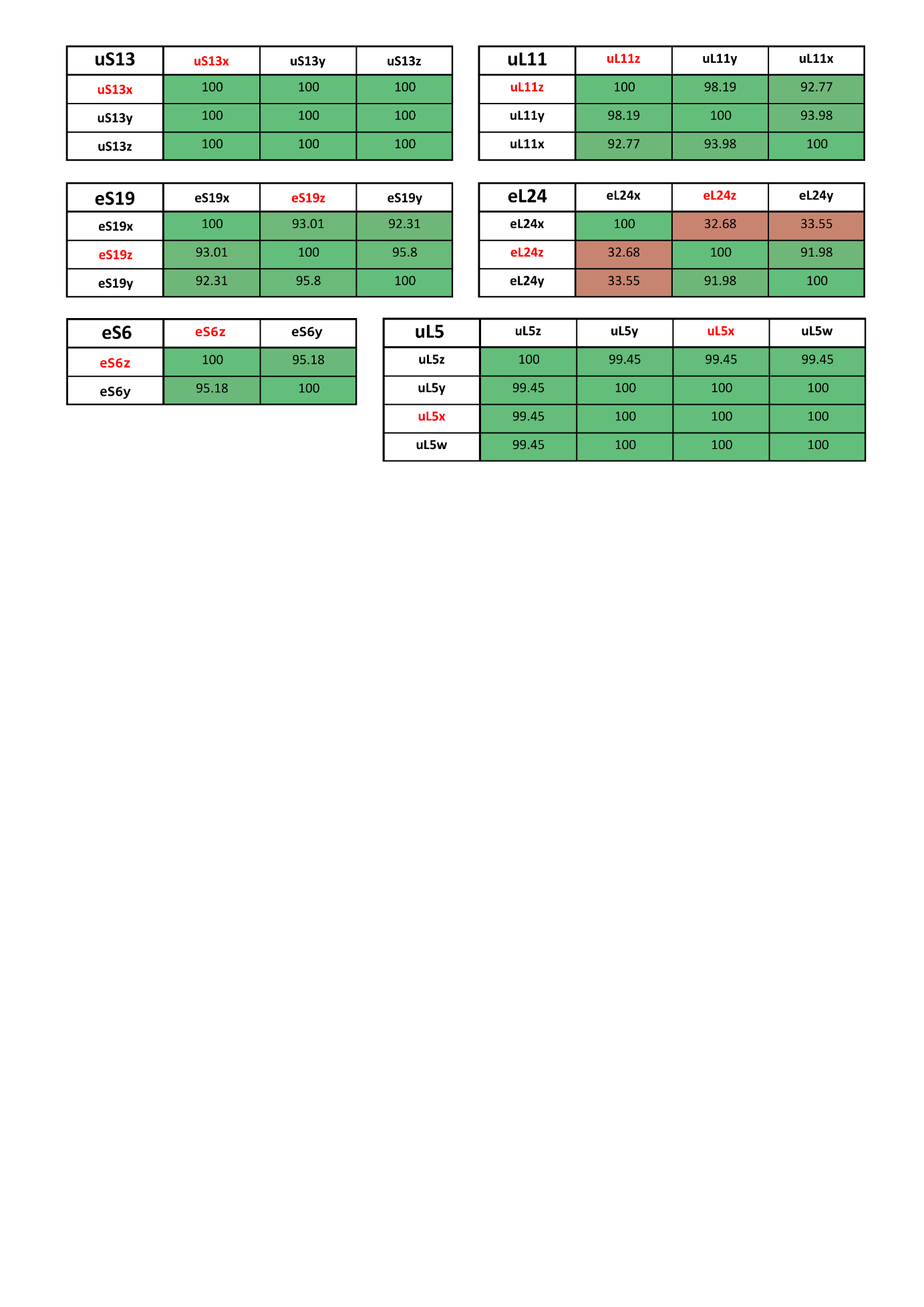
**

**Supplementary Figure 3: RP paralogues sequence identity and paralogue selection**

Tables of sequence identity matrix for the *Arabidopsis thaliana* RP paralogue genes are shown in the upper part. Protein sequences were obtained from the TAIR database at the respective AGI code and aligned with the MUSCLE algorithm. The colour of the box represents the percentage of sequence identity (gradient from low red to high green indicates increasing % identity). Selected genes for cloning are highlighted in red.

**
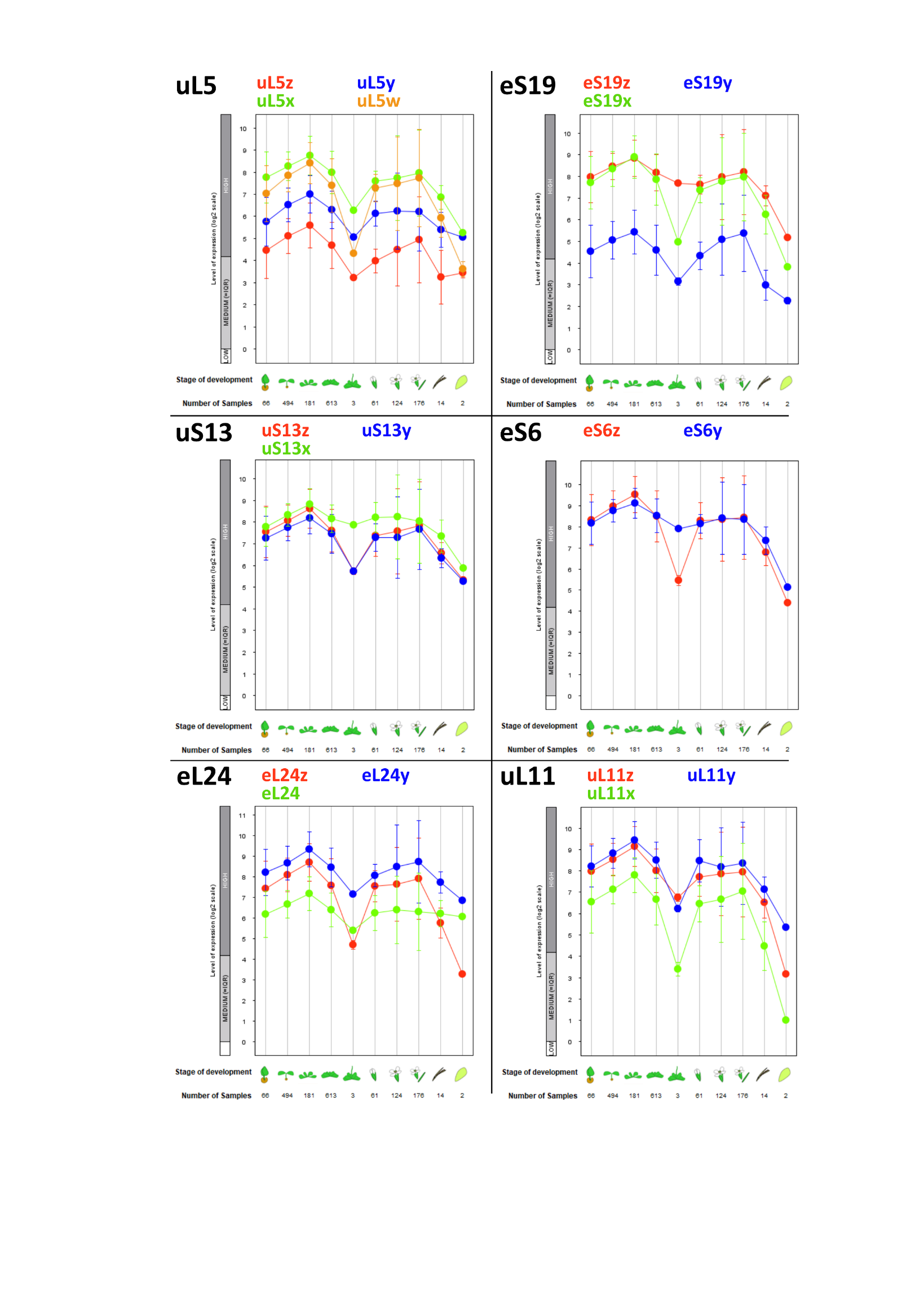
**

**Supplementary Figure 4: RP paralogues expression profiles**

The expression profiles for the *Arabidopsis thaliana* RPs paralogue genes are displayed according to Affymetrix GeneChip data and were generated using the Development functions of Genevestigator®. The expression profiles are shown for uL5x (upper left), uS13x (middle left), eL24z (lower left), eS19z (upper right), eS6z (middle right), and uL11z (lower right). Data from ATH arrays are presented in scatter plot diagrams. The x-axis represents the following developmental stages, from left to right: germinating seed, seedling, young rosette, developed rosette, bolting, young flower, developed flower, flowers and siliques, mature siliques, and senescent leaves. For each data point, the number of samples is indicated. The values in the plots are the mean values, and the error bars show standard errors.

**
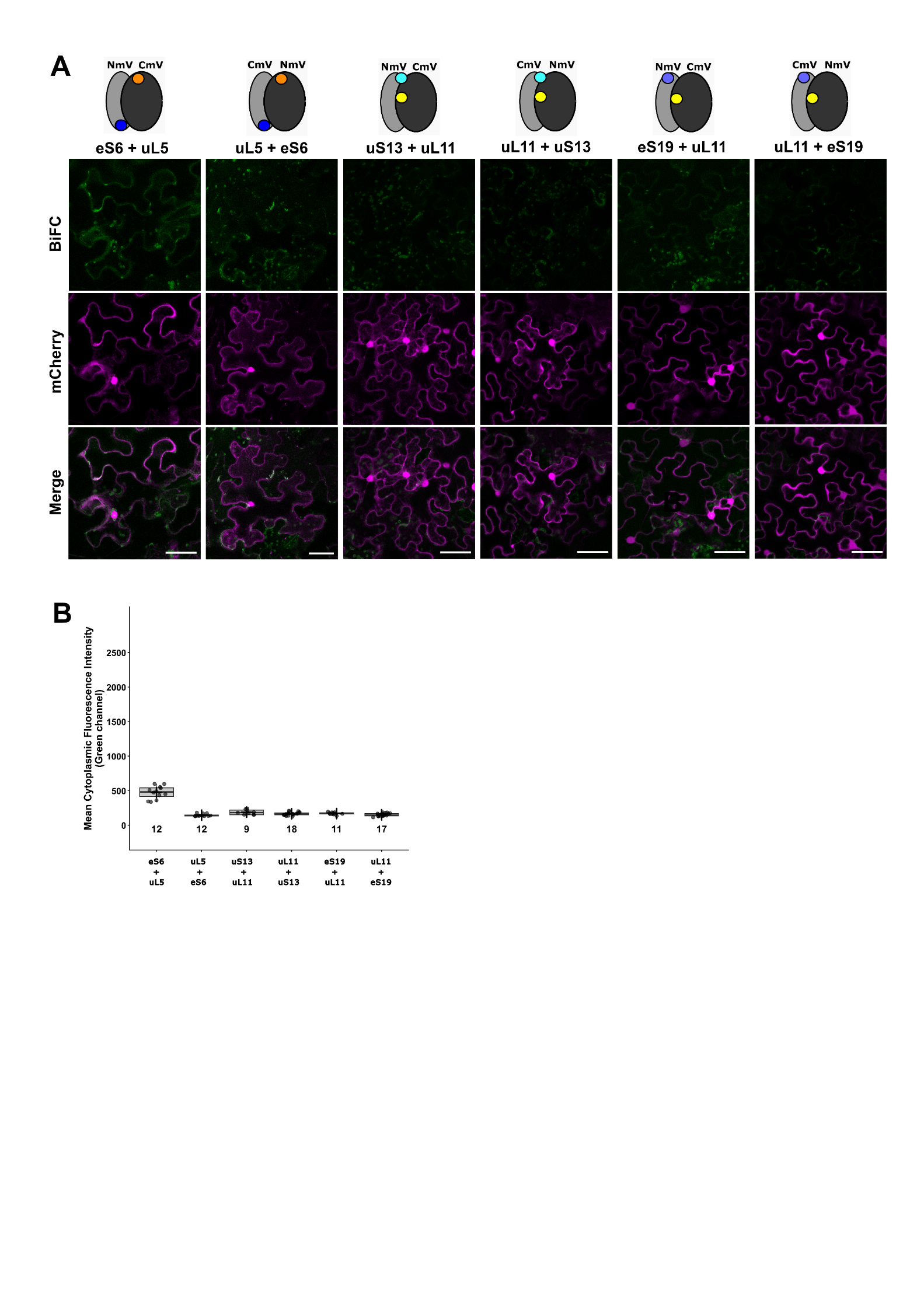
Supplementary Figure 5: Suboptimal Ribo-BiFC combinations in *Nicotiana* *benthamiana***

**(A)** Transient *N.* *benthamiana* assay of Ribo-BiFC reciprocal combinations in suboptimal distance. The upper cartoon section depicts the position and C-terminal tagging with the mVenus BiFC fragment. Representative images of the signal detection are shown in the green channel for mVenus BiFC. Free mCherry control of the pavement cells' transient transformation is in the middle (shown in magenta) and the overlay of the channels is displayed in the third row. All transcription units are driven by the pCsVMV promoter and acquired channels were processed in the same way. Scale bars equal to 50 μm. **(B)** Ribo-BiFC quantification of mean cytoplasmic fluorescence intensity. Boxplots show median (center line), interquartile range (box), and whiskers. Individual data points are overlaid, mean values indicated by +. Numbers indicate sample size (n).


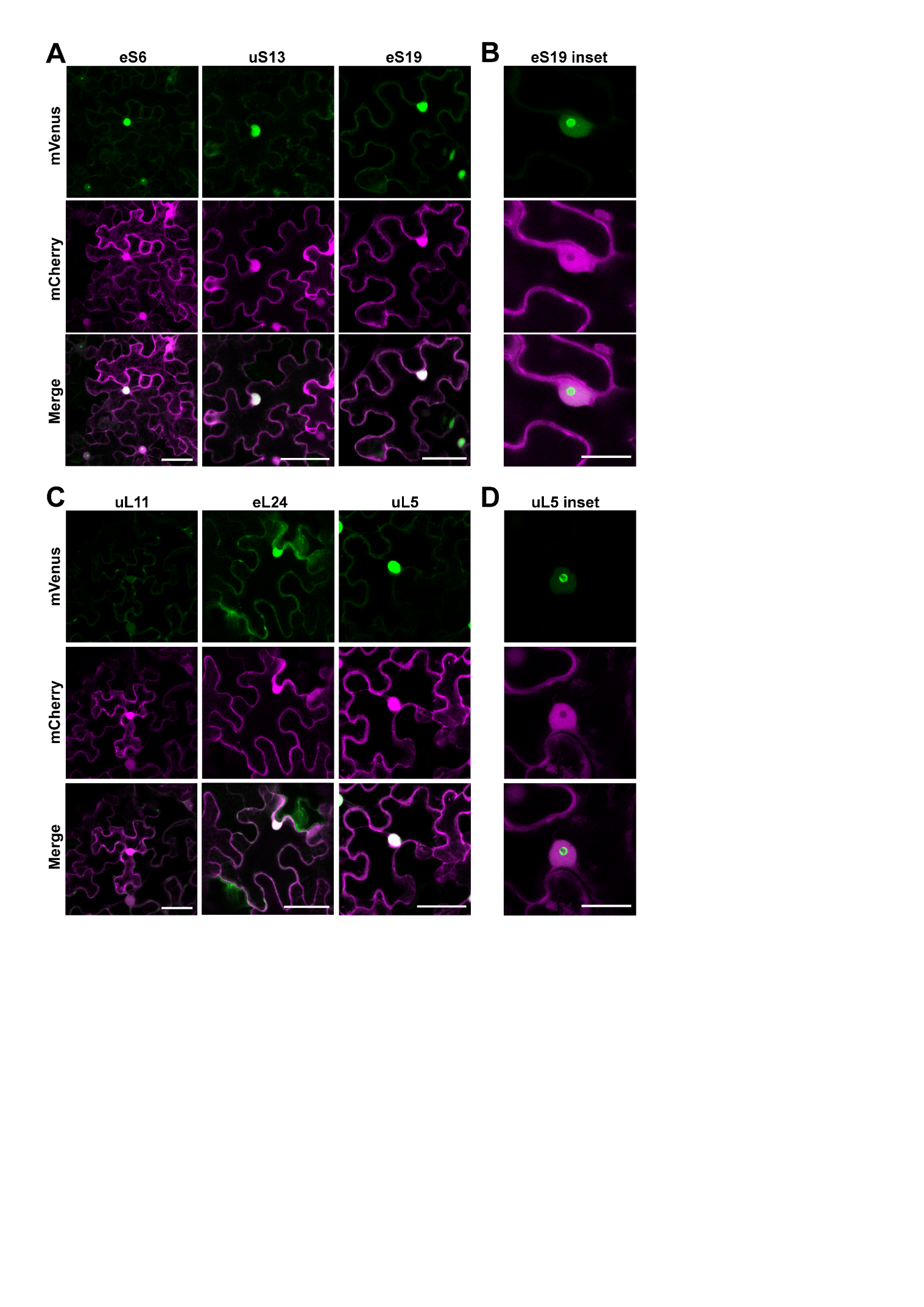
Supplementary Figure 6: Subcellular localization of mVenus tagged proteins in N. benthamiana.

Selected proteins were fused to complete mVenus sequence and served as controls to Ribo-BiFC experiments. **(A,C)** For each sample, the protein-specific localization is displayed in the green channel (mVenus) and transformation control in magenta (free mCherry). The merge of the channels is shown at the bottom. Scale bar equals to 50 μm. **(B,D)** Nuclear and nucleolar localizations of the RPs are represented by eS19 and uL5 insets in the green channel. All protein fusions are driven by the pCsVMV promoter. Scale bars equal to 20 μm.

**
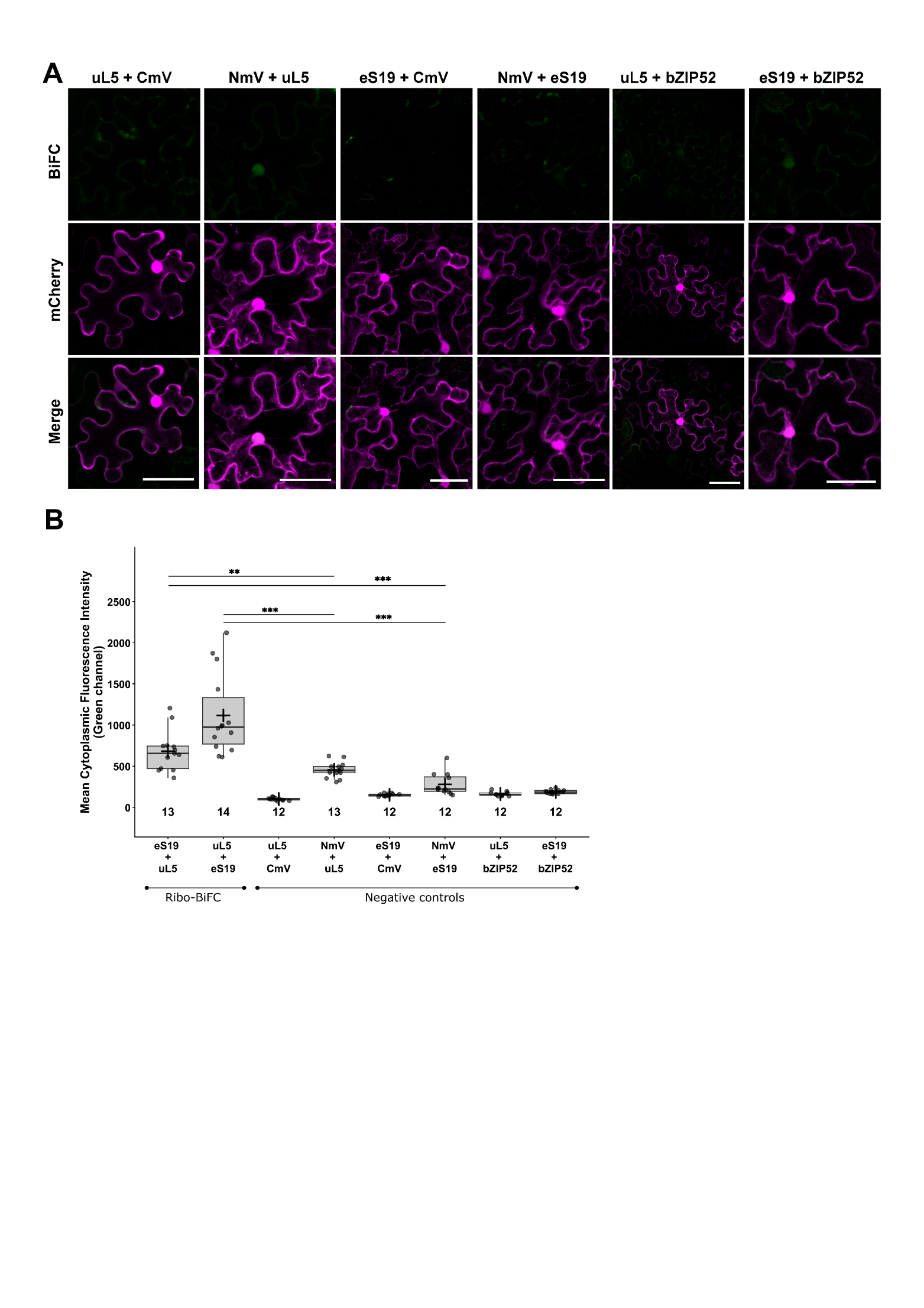
**

**Supplementary Figure 7: Ribo-BiFC negative controls in pavement cells of *N. benthamiana***

**(A**) For the transient tobacco assay, non-interacting partners were selected (upper row). The samples of negative controls include protein-protein combinations, RPs and bZIP52 fused to NmV or CmV, followed by protein-split mVenus pairs and RPs with free BiFC complementing fragments (free NmV or free CmV). Constructs are driven by pCsVMV promoter, co-expressed with free mCherry and co-infiltrated with P19 silencing suppressor. Scale bars equal to 50 μm. **(B)** Mean cytoplasmic fluorescence intensity of the additional BiFC controls with the values for the uL5 and eS19 Ribo-BiFC already presented as part of the Figure 2D for comparison. Boxplots show median (center line), interquartile range (box), and whiskers. Individual data points are overlaid, mean values indicated by +. Numbers indicate sample size (n). Significant differences between the Ribo-BiFC and controls showing cytoplasmic signal were determined by unpaired two-tailed Mann-Whitney U tests (** p < 0.01, *** p < 0.001).

**Supplementary Table 1: Sequences of RPs for domestication**


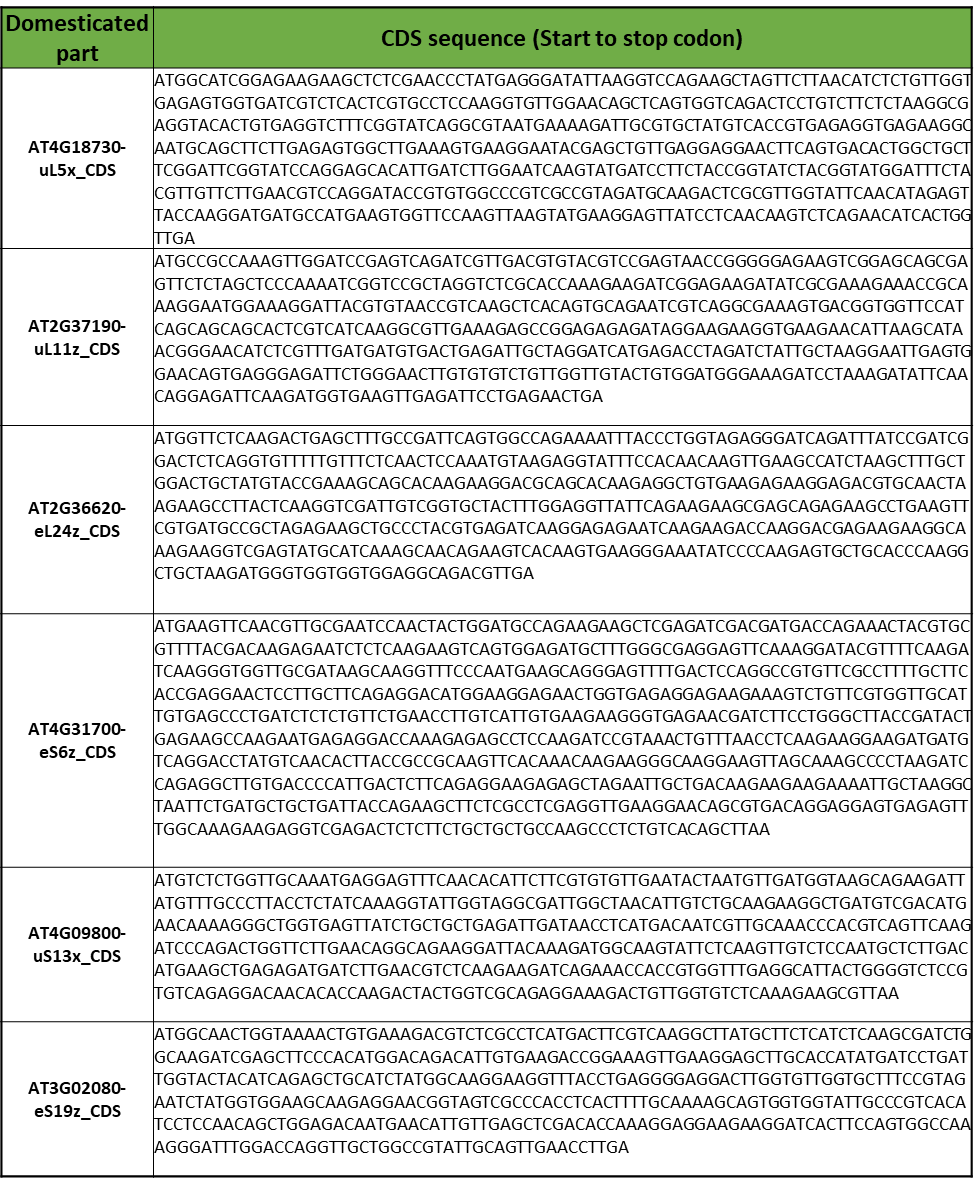


**Supplementary Table 2: Sequences of BiFC fragments**


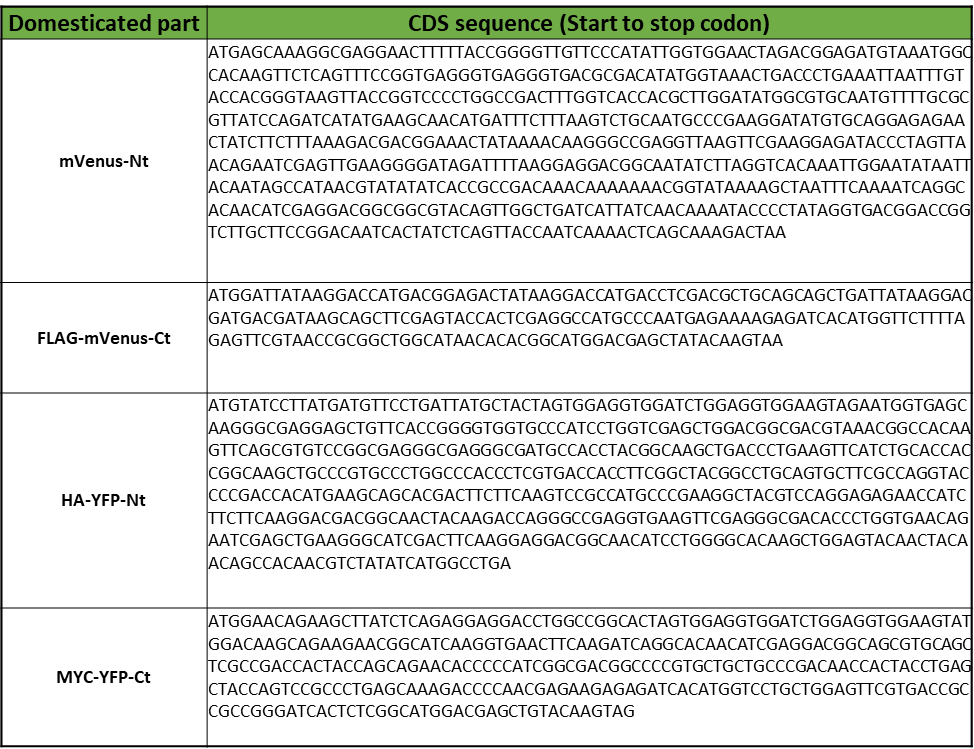


**Supplementary Table 3: Oligonucleotide sequences for domestication of RPs or BiFC**


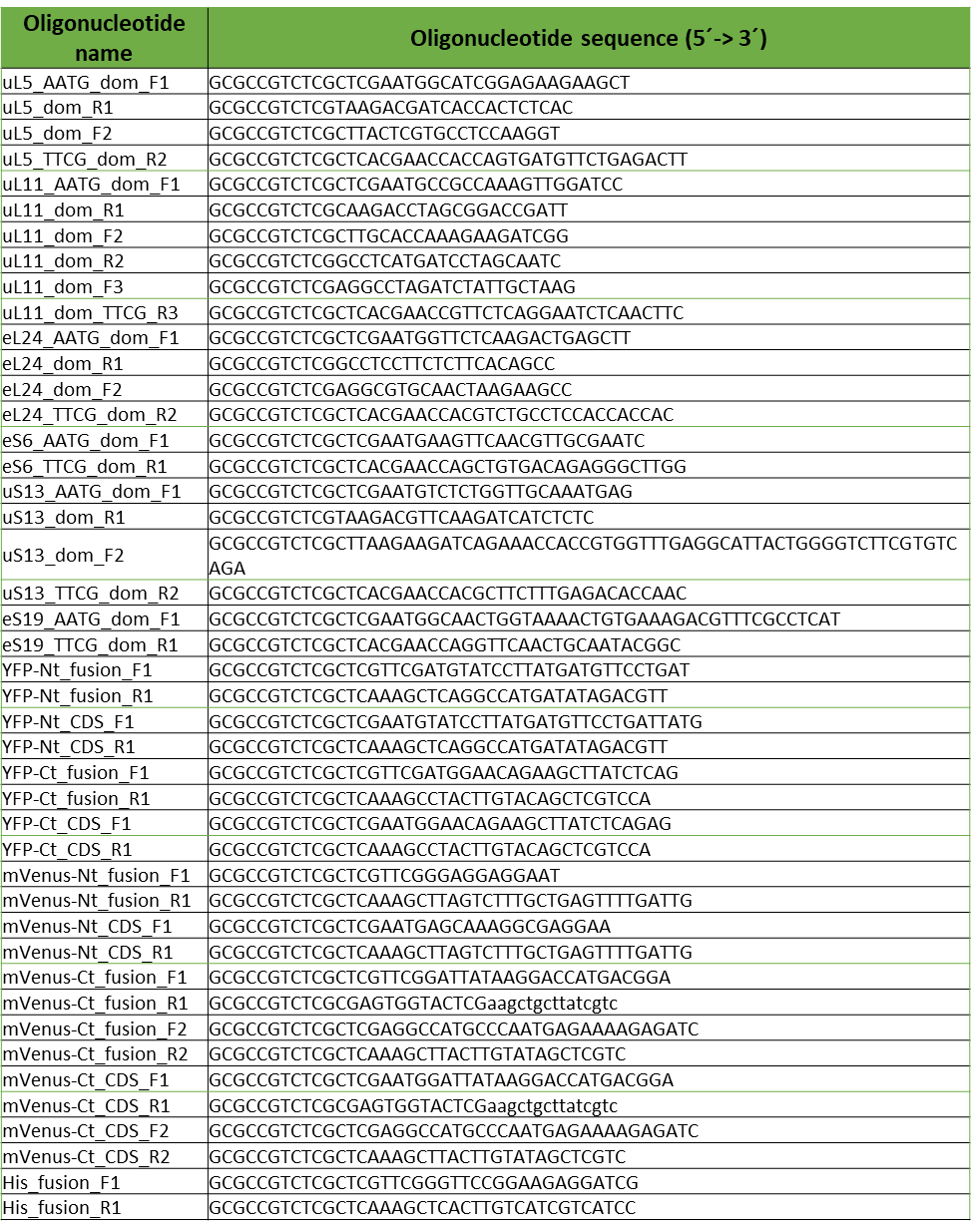

Supplement: Supplementary file 1 — Supplementary Material 1. [file 13007_2025_1494_MOESM1_ESM.docx]
